# Preferences of people with mental illness for engaging in exercise programs under COVID-19 restrictions

**DOI:** 10.1177/1039856220975299

**Published:** 2020-12-30

**Authors:** Justin J Chapman, Emily Hielscher, Sue Patterson, Nicola Reavley, Wendy J Brown, Marianne Wyder, Sarah Childs, Anneliese Russell, Shuichi Suetani, James G Scott

**Affiliations:** QIMR Berghofer Medical Research Institute, Australia; Metro South Addiction and Mental Health Service, Australia; Queensland Police-Citizens Youth Welfare Association, Australia; School of Human Movement and Nutrition Sciences, The University of Queensland, Australia; QIMR Berghofer Medical Research Institute, Australia; Metro North Mental Health Service, Australia; University of Melbourne, Australia; School of Human Movement and Nutrition Sciences, The University of Queensland, Australia; Metro South Addiction and Mental Health Service, Australia; Queensland Alliance for Mental Health, Australia; Richmond Fellowship Queensland, Australia; Metro South Addiction and Mental Health Service, Australia; School of Medicine, Griffith University, Australia; School of Medicine, The University of Queensland, Australia; QIMR Berghofer Medical Research Institute, Australia; Metro North Mental Health Service, Australia; School of Medicine, The University of Queensland, Australia

**Keywords:** physical activity, preferences, intervention design, mental health, exercise

## Abstract

**Objectives:**

People with mental illness may be vulnerable to decline in mental health and reduced physical activity because of the COVID-19 pandemic and associated restrictions. The aim of this study was to inform the design of physical activity interventions for implementation under these conditions to improve/maintain well-being and physical activity in this population.

**Methods:**

People with mental illness who had participated in a physical activity program prior to the pandemic were invited to complete a survey about the impact of COVID-19 on mental health and physical activity and their preferences for engaging in a physical activity program under pandemic-related restrictions.

**Results:**

More than half the 59 respondents reported worse mental health and lower physical activity during the pandemic. The preferred format for a physical activity program was one-on-one exercise instruction in-person in a park. Program components endorsed as helpful included incentivization, provision of exercise equipment and fitness devices, and daily exercise programs. About a third of the participants reported limitations in using technology for a physical activity program.

**Conclusions:**

In-person exercise support is preferred by people with mental illnesses during pandemic-related restrictions. Enablement strategies such as providing equipment and self-monitoring devices should be utilized; assistance may be needed to incorporate the use of technology in exercise programs.

The physical and mental health benefits of physical activity (PA) are widely recognized.^1^ The coronavirus disease 2019 (COVID-19) pandemic and associated government-imposed restrictions designed to contain the spread of the virus have resulted in substantial decreases in PA globally,^2^ with negative impacts on the mental health and well-being of the population.^3^ People with mental illness may be particularly vulnerable; a recent study reported that distress was heightened in people with mood disorders compared with people without mental illness, and that distress was highest in people who reported lower PA during the pandemic.^4^ PA can improve symptoms of many mental illnesses,^5^ and supporting people with mental illness to maintain PA through the pandemic is essential to promote physical and mental well-being and prevent widening of health inequalities.^6^

PA interventions can be adapted for delivery under pandemic-related restrictions by using telehealth services or other methods; however, little is known about their design and implementation for people with mental illness under such conditions. It is foreseeable that until a vaccine is available, Australia will be subject to fluctuating community restrictions disruptive to usual program delivery. This study aimed to inform exercise program design and implementation for people with mental illness during pandemic-related restrictions. The primary objective was to profile mental health, changes in PA levels, access to resources for exercise programs, and preferences and opinions for engaging in PA programs under pandemic-related restrictions.

## Methods

This was a questionnaire-based cross-sectional study of people with mental illness who had, prior to the pandemic, participated in a community exercise program. Ethical approval was obtained from the QIMR Berghofer Medical Research Human Research Ethics Committee (P3616).

### Procedure

Potentially eligible individuals were identified from a register of participants of community-based lifestyle programs at the Queensland Police-Citizens Youth Welfare Association in Australia. Participants of these programs were (i) aged over 18 years and (ii) referred to the program from public mental health services, mental health non-government organizations, and private or general practice clinics. The lead author (JC) contacted potentially eligible individuals, and emailed or text messaged the link to the online survey to those who agreed to participate. Completing the survey implied consent for participation. Participants were offered a $20 gift card for completing the survey. Survey responses were received in May 2020, during which time outdoor gatherings of up to 10 people were permitted in Queensland, Australia (reinstated 15 May).

### Measures

Self-reported sociodemographic (age, sex, education, employment status, financial management, Aboriginal and Torres Strait Islander identification), psychosocial (psychological distress and loneliness), and health information (mental and physical health diagnoses assessed using multi-choice and open-text response), and information on usual PA *before* and *after* COVID-19 were collected. Outcome measures are outlined in Table 1.

**Table 1.** Overview of outcomes measures

***Impact of COVID-19***: The impact of the COVID-19 pandemic and associated restrictions on mental health was assessed using the question: *How has your mental health and well-being been affected by COVID-19 pandemic and associated restrictions?*; responses on a five-point scale (*much worse, somewhat worse, about the same, somewhat better, much better*)
***K6***: The six-item K6 was used to assess general psychological distress experienced in the past month using a five-point scale; scores over 15 indicate high distress^7^
***Three-item loneliness scale***: Participants were asked how often they feel (i) that they lack companionship, (ii) left out, and (iii) isolated from others; responses on a three-point scale (*hardly ever, some of the time, often*)^8^
***4-PAQ*:** The 4-PAQ was adapted from the PAVS questions^9^ which ask about physical activity completed in the previous week. The 4-PAQ was adapted to ask about *average* exercise participation rather than previous week and to distinguish high-intensity exercise. PA questions specified time periods before and after “COVID-19 and associated restrictions”
***Exercise preferences and opinions*:** To assess preference for the format of an exercise program, participants were asked: *If you had a free exercise program available during current COVID-19 restrictions, what format would you like?* To assess opinions on what would be helpful components of such an exercise program (e.g. use of diaries, fitness trackers, incentives, etc.), participants were asked: *If you had a free exercise program available during current COVID-19 restrictions, what would you find helpful?*
***Resource access and self-efficacy*:** Access to resources (technology and exercise related) was assessed using 11 items, with yes/no responses. As a measure of self-efficacy, participants were also asked about their confidence to use technology (to use online videos, video messaging services, phone apps), and confidence in exercising on their own, or if having a bad day

*Note.* 4-PAQ = four-item physical activity questionnaire, K6 = Kessler-6 scale of psychological distress, PAVS = Physical Activity as a Vital Sign.

### Analysis

Summary statistics are presented for all measures. Estimates for weekly exercise were calculated by multiplying the number of reported exercise days by the duration of exercise and dichotomized into *sufficiently* or *insufficiently* active (exercising more or less than 150 min/week, respectively). A related-samples McNemar test was used to test for differences in PA participation before and during COVID-19. All analyses were conducted in SPSS v23.

## Results

### Participants

Most of the 59 respondents (58%) reported that their mental health was negatively affected by COVID-19, with about one-fifth (19%) reporting *much worse* mental health. Most (*n* = 40; 68%) had high psychological distress (mean = 17.4; standard deviation [SD] = 6.11), and one-third (34%) had the highest loneliness rating. Health and demographic statistics are shown in Table 2.

**Table 2.** Baseline health and demographic characteristics (N = 59)

	Mean	(SD)
**Age, years**	43.1	(14.5)
* Missing* (*n* = 3)		
	*n*	(%)
**Sex**	** **	** **
Female	44	75%
Male	11	19%
Prefer to self-identify	1	2%
* Missing*	*3*	5%
**Aboriginal or Torres Strait Islander**	** **	** **
Aboriginal and/or Torres Strait Islander	5	8%
Neither	49	83%
* Missing*	*5*	*8%*
**Education**	** **	** **
University degree	9	15%
Trade/apprenticeship	8	14%
Diploma	11	19%
High school	17	29%
Did not finish high school	11	19%
*Missing*	*3*	*5%*
**Employment**	** **	** **
Employed full-time	2	3%
Employed part-time (less than 35 h/week)	8	14%
Employed casually	6	10%
Unemployed and looking for work	6	10%
Student/volunteer/homemaker	6	10%
Retired	5	8%
Unable to work due to mental or physical health	22	37%
* Missing*	*4*	*7%*
**Self-reported mental health diagnoses**		
*Number of diagnoses*		
None	9	15%
One	12	20%
Two or more	34	58%
* Missing*	4	7%
*Diagnoses* ^a^		
Depression	32	54%
Anxiety disorder	30	51%
Trauma-related disorder	16	27%
Psychotic disorder	11	19%
Bipolar disorder	10	17%
Personality spectrum disorder	8	14%
Substance use disorder	4	7%
Eating disorder	6	10%
Other (e.g. Attention deficit hyperactivity disorder, autism, adjustment, disorder)	6	10%
**Self-reported physical health condition**		
*Number of conditions*		
No conditions reported	27	46%
One or more condition reported	*33*	54%
*Conditions*		
Asthma	*11*	*26%*
Rheumatoid or osteoarthritis	*9*	*21%*
Cardiovascular disease	*5*	*12%*
Diabetes (type 1 or 2)	*4*	*10%*
Chronic bronchitis, emphysema, or other pulmonary condition	*3*	*7%*
Osteoporosis	*3*	*7%*
Neurological (multiple sclerosis, Parkinson’s, stroke, Alzheimer’s, dementia, other)	*3*	*7%*
Liver disease/condition	*2*	*5%*
Cancer (other than non-melanoma skin cancer)	*1*	*2%*
Kidney disease/condition	*1*	*2%*

a All diagnoses are listed, including for those who reported multiple diagnoses; hence, the proportions sum to greater than 100%.

A total of 30 (51%) respondents reported exercising at least 150 min/week before COVID-19, which reduced to 12 (20%) during COVID-19. High-intensity exercise was reported by 32 participants before COVID-19 (mean = 107 min/week; SD = 149 min/week), and by 21 participants since COVID-19 (mean = 41 min/week; SD = 68 min/week). A related-samples McNemar test confirmed that the reduction in participants exercising at least 150 min/week and those doing any high-intensity exercise was statistically significant (*p* = .001 and *p* = .013, respectively).

### Access to resources

When asked about access to equipment that could be used for PA, most respondents reported *not* having a pedometer (80%), fitness tracker (76%), or equipment for aerobic (76%) or resistance exercise (63%); however, most said they have enough space for home-based exercise (63%). A high proportion of respondents reported having a phone (95%) or smartphone (90%); fewer reported having a computer (68%) or tablet (44%). However, only 63% reported having an internet connection suitable for supporting video streaming or calls (85% had internet connection, 74% of whom had a *suitable* internet connection).

### Exercise preferences and opinions

A high proportion of respondents *disagreed* that they were as active as they would like to be (83%). Reported self-efficacy for exercise was low: 64% *disagreed* that they would be confident exercising on their own and 76% disagreed that they could make themselves exercise if having a bad day. When asked about confidence in using online mediums for exercise support, about a quarter of those with an internet connection reported being *not at all confident* in their ability to access exercise videos (26%), use video messaging services (24%), or use smartphone applications related to exercise (30%).

In relation to exercise program formats, one-on-one exercise instruction outdoors in a park was the most preferred (81%); other formats were endorsed by fewer than 40% of participants. The most disliked suggestions were one-on-one exercise instruction via video and group educational sessions over phone or video (41% and 42%, respectively); results are provided in Table 3. Program components endorsed as helpful included: incentives (e.g. gift cards; 73%), having home exercise equipment delivered (69%), the use of fitness devices (e.g. monitors and trackers; 63%), use of phone apps (56%), and having daily exercise programs sent to them (54%). The involvement of social media had the highest *unhelpful* rating (34% rated unhelpful); results are provided in Table 4.

**Table 3.** Preferred format for exercise program (*N* = 59)

	*Dislike*	*Neutral*	*Like*
One-on-one exercise instruction *in-person*, while you exercise outdoors in a park	3 (5%)	7 (12%)	48 (81%)
One-on-one exercise instruction via *video*, while you exercise	24 (41%)	18 (31%)	17 (29%)
One-on-one exercise *consultation* (e.g. advice and goal setting) over phone or video	17 (29%)	19 (32%)	23 (39%)
Group educational sessions over phone or video	25 (42%)	18 (31%)	16 (27%)
An online community for group support and chat about exercise	17 (29%)	21 (36%)	21 (36%)

**Table 4.** Helpful program components (*N* = 59)

	Unhelpful	Neutral	Helpful
Providing incentives (e.g. gift cards)	2 (3%)	14 (24%)	43 (73%)
Home exercise equipment delivered to my door	8 (14%)	10 (17%)	41 (69%)
Use of fitness devices for self-monitoring (e.g. HR monitors, fitness trackers)	5 (8%)	16 (27%)	37 (63%)
Use of phone apps for tracking progress	7 (12%)	19 (32%)	33 (56%)
Daily exercise programs emailed/texted to me	12 (20%)	15 (25%)	32 (54%)
Video workouts to follow along with (e.g. on YouTube)	11 (19%)	21 (36%)	27 (46%)
Use of diaries for tracking progress	8 (14%)	23 (39%)	27 (46%)
Competition with other participants (e.g. step count leader board)	9 (15%)	24 (41%)	26 (44%)
Include information related to COVID-19 and maintaining health and well-being	8 (14%)	23 (39%)	26 (44%)
Involvement of other social media platforms (e.g. Facebook, Microsoft Teams, Instagram)	20 (34%)	18 (31%)	21 (36%)

## Discussion

The COVID-19 pandemic has had a major impact on society. Participants in this study had high psychological distress and loneliness and reported worse mental health and lower PA during the pandemic. Limited access to resources must be considered in design of PA interventions. Relatively low access to exercise equipment and a suitable internet service in this group limits the potential for remote interventions (e.g. telehealth) to enable home-based exercise. Interventions employing online mediums for people with appropriate internet access should include assistance to establish the service (e.g. set up user accounts) and navigate applications; however, other mediums are required to reach people with unsuitable internet access, which may be pertinent to rural communities. Participants perceived use of fitness trackers or phone apps for self-monitoring as helpful program components; however, low ownership of fitness devices and low confidence in using technology imply that more practical support would be required to incorporate these components. This is consistent with a recent survey study demonstrating high smartphone ownership but low app usage in people with mental illness,^10^ and that m-Health and wearable technologies are feasible when ongoing individual technical assistance is provided.^11^

Practical support and enablement strategies were preferred, consistent with research related to exercise preferences during non-pandemic conditions.^12^ Although internet or phone formats would arguably reduce chances of virus transmission, participants still prefer in-person support such as one-on-one exercise instruction. Education strategies to encourage family or carers of people with mental illness to provide in-person exercise support (e.g. accompaniment while walking) could be effective. Helpful program components included incentives (e.g. gift cards), home exercise equipment delivered, use of fitness devices or phone apps for self-monitoring, and daily exercise programs sent to them. Providing home exercise equipment combined with prescribed exercise programs to utilize the equipment could enable home-based exercise and facilitate skill building.

Limitations of this research are a small sample that limits generalizability and lack of clinically confirmed diagnoses or other information relevant to condition (e.g. functioning, stage of illness). Participants were recruited from a register of previous exercise participants with the view of informing program adaptation for pandemic-related restrictions.

## Conclusions

People with mental illness may be vulnerable to decline in mental health and lower levels of PA because of the COVID-19 pandemic and associated restrictions. Members of this group want to be more active and prefer in-person exercise support during government-imposed restrictions. Participation in PA can be promoted by incentivization, enablement via provisions of equipment and professional instruction, and self-monitoring strategies. A high proportion of people with mental illness own smartphones, which could be utilized in exercise interventions for self-monitoring or exercise instruction; however, there may be problems with internet limitations, and some may need additional support because of low confidence in using this medium.
